# Graphene Nanoplatelets Modified with Amino-Groups by Ultrasonic Radiation of Variable Frequency for Potential Adsorption of Uremic Toxins

**DOI:** 10.3390/nano9091261

**Published:** 2019-09-05

**Authors:** C. Cabello-Alvarado, M. Andrade-Guel, M. Pérez-Alvarez, G. Cadenas-Pliego, Dora A. Cortés-Hernández, P. Bartolo-Pérez, C.A. Ávila-Orta, V.J. Cruz-Delgado, A. Zepeda-Pedreguera

**Affiliations:** 1 CONACYT Research Fellow-Research and Innovation Consortium of the State of Tlaxcala, C.P. 90000 Tlaxcala, Mexico; 2 Center for Research in Applied Chemistry (CIQA), Saltillo, 25315 Coahuila, México; 3 CONACYT Research Fellow-Mexican Petroleum Institute, Eje Central Lázaro Cárdenas Norte 152, 07730 Ciudad de México, México; 4 Center for Research and Advanced Studies of the National Polytechnic Institute (CINVESTAV) Saltillo Unit. Av. Industria Metalúrgica #1062 Parque Industrial Saltillo-Ramos Arizpe, 25900 Saltillo, México; 5 Center for Research and Advanced Studies of the National Polytechnic Institute (CINVESTAV) Mérida Unit, Km. 6 Antigua Carretera a Progreso, Apartado postal 73 Cordemex, Mérida, 97310 Yucatán, México; 6 CONACYT Research Fellow- Materials Unit, Yucatan Scientific Research Center, A.C., 97205 Mérida, México; 7 Autonomous University of Yucatan(UADY), Mérida, 97310 Yucatán, México

**Keywords:** ultrasound, surface modification, adsorption, uremic toxins

## Abstract

Chronic kidney disease (CKD) is a worldwide public health problem. In stages III and IV of CKD, uremic toxins must be removed from the patient by absorption, through a treatment commonly called hemodialysis. Aiming to improve the absorption of uremic toxins, we have studied its absorption in chemically modified graphene nanoplatelets (GNPs). This study involved the reaction between GNPs and diamines with reaction times of 30, 45 and 60 min using ultrasound waves of different amplitudes and frequencies. Functionalized GNPs were analyzed by Fourier Fourier-transform infrared spectroscopy (FTIR), X-ray photoelectron spectroscopy (XPS), Scanning electron microscopy and energy dispersitive spectroscopy (SEM-EDS), and Thermogravimetric analysis (TGA). The analysis of the functional groups confirmed the presence of amide and hydroxyl groups on the surface of the GNPs by reactions of diamines with carboxylic acids and epoxides. Adsorption of uremic toxins was determined using equilibrium isotherms, where the maximum percentage of removal of uremic toxins was 97%. Dispersion of modified graphene nanoplatelets was evaluated in water, ethanol and hexane, as a result of this treatment was achieved a good and effective dispersion of diamines-modified graphene nanoplatelets in ethanol and hexane. Finally, the results of hemolysis assays of the modified graphene with amine demonstrated that it was not cytotoxic when using 500 mg/mL. The samples of modified graphene demonstrated low degree of hemolysis (<2%), so this material can be used for in vivo applications such as hemodialysis.

## 1. Introduction

A common health problem in different countries is chronic kidney disease (CKD), which has an increasing incidence and prevalence, and high cost of treatment. The main issue is that kidneys do not fulfill their function of purifying the blood. This illness leads to the accumulation of toxic molecules that are normally eliminated by healthy kidneys. These toxic molecules are known as uremic toxins, among which are uric acid, urea and creatinine, which are insoluble in water, and their removal is difficult due to their low molecular weight. To solve this problem, the patient is treated with hemodialysis with non-selective membranes, which are not completely efficient and present high cost for patients [1]. The hemodialysis process requires materials with various characteristics, including: high capability to eliminate uremic toxins indiscriminately, light weight, low cost, good chemical stability, high hemocompatibility and good reusability.

High levels of uremic toxins, such as uric acid and urea in the blood, can lead to the formation of solid crystals that locate in the joints, causing episodes of acute pain, in affected people. If high levels of uric acid and urea persist in the blood, they can cause cell death and even reach the stage of cancer [2]. One of the main ways to solve high levels of uric acid and urea is the adsorption of this type of toxin in a good and selective adsorbent.

Nanoporous adsorbents with high surface area have offered a great potential as biomaterials for CKD. There are several different types of adsorbents, including activated carbon, resins, carbon nanotubes, mesoporous silica, zeolites, metal-organic frameworks, etc.

Adsorbents from carbon-based materials (carbon nanomaterials) are some of the most promising materials for various applications, due to their high performance and relatively low cost. These materials have demonstrated high capability of adsorption for different organic and inorganic compounds and heavy metal ions [3,4,5].

Among the carbon-based materials, nanotubes, nanofibers and graphene sheets of micrometric and nanometric dimensions have been studied as adsorbents, due to their size and high specific surface area. Graphene is a carbon-based material that can be found in different arrangements, such as: graphene monolayers, graphene nanosheets and graphene nanoplatelets (GNPs) [6].

GNPs are a low-cost alternative in comparison to graphene nanosheets or monolayer graphene, since the latter have higher prices. Their theoretical high specific surface area (2630 m^2^/g), compared to that of carbon nanotubes (1315 m^2^/g), makes GNPs an attractive candidate for adsorption applications [7,8].

Hemolysis is another topic related to the hemocompatibility of biomaterials in contact with blood, the hemolysis ratio is an important parameter for evaluating blood compatibility. The lower the hemolysis rate, the better the blood compatibility of the material, and there have recently been some reports of carbon nanomaterials with excellent hemocompatibility [9,10,11,12] and high adsorption capacity [13,14].

Chemical modification of graphene produces interfacial interactions with organic molecules, especially with those containing nitrogen atoms, leading to covalent bond formation between carbon and nitrogen, helping to promote selective adsorption. Modification of carbon-based particles is most often carried out by means of a strong treatment, with a mixture of sulfuric and nitric acids or mixture of strong oxidizing agents such as sulfuric acid and potassium permanganate [15,16,17]. However, the uses of these chemical reagents affect the environment and the structure of the platelets due to their aggressiveness and toxicity. GNPs contain in their chemical structure carboxylic groups, which may react with amines into reaction conditions that no generated secondary harmful effects to environment neither for laboratory personal.

One of the most promising modification methods is sonication or ultrasonic radiation, this technique supports green chemistry because it decreases reaction times, also direct more selective synthetic processes favoring the formation of the desired products, and reduces the wastes. The effect of high intensity ultrasound vibration at specific or different frequencies has been used in different types of materials, since it is very effective activating surfaces of different particles [18,19]. This technique involves the use of ultrasonic radiation. The acoustic energy tends to favor stable dispersions in colloidal solutions and even in nanoparticles. The effect of ultrasonic radiation in a solution is directly related to the phenomenon of cavitation, generating active sites and high concentration of shear stresses [20,21]. The modification and dispersion of graphene using ultrasonic waves of different amplitudes and frequencies is an excellent strategy, with the energy generated favoring the chemical reactions; in general, this method is more effective compared with conventional methods. The advantage of using ultrasonic probe is that it is applied directly in the liquid medium, where the chemical reaction between the interface of the material and the modification of the molecules can be carried out. This is more difficult using an ultrasound bath, because the solution must be inside a glass container, which is a limitation for the acoustic waves acting on the liquid medium. Ultrasound energy produces cavitation and shear forces, which could generate high pressure and immense heat in a specific area in a very short period of time; we are talking around 500 atm and 5000 °C [22]. Another advantage of the sonication process is that it helps to deagglomerate nano-sized particles, exposing the whole surface area where there may be a chemical interaction [23]. In our research group, the technique of ultrasound radiation has been applied successfully to modify carbon nanotubes with amines and carboxylic acids [24,25].

In this work, we report the modification of GNPs with a mixture of 1,4-diaminobutane dihydrochloride and 1,3-diaminopropane by ultrasonic waves of different amplitudes and frequencies in aqueous medium, for their application in the adsorption of uremic toxins. Selection of amines was performed according their origin; 1,4-Diaminobutane is a biogenic amine that is formed during the decomposition processes of organic matter, which gives it the characteristic foul odor (putrescine) and 1,3-diaminopropane is one of the compounds found in urinary excretion [26,27]. We suppose that a combination or mixture of these amines could be more effective for chemical modification, than their use separately. The presence of amino groups on the surface of GNPs should favor the adsorption of uremic toxins. 

## 2. Materials and Methods

### 2.1. Reagents and Materials

The nanoparticles used were industrial grade graphene nanoplatelets (10–12 layers) with 97% purity and diameter of 2 to 3 μm from Cheap Tubes, Inc. (Cambridgeport, VT, USA). A mixture of two amines, 1,4-diaminobutane dihydrochloride and 1,3-diaminopropane, both with 99% purity (Sigma Aldrich, Saint Louis, MO, USA) in 1:1 ratio (w/w), was used. Distilled water with a pH of 7 was used as a solvent to obtain the concentrated aqueous solutions with the two modifying reagents. Ethanol (99.5%) and hexane anhydrous (99.0%) were also used as solvents (Sigma Aldrich, Saint Louis, MO, USA).

### 2.2. Chemical Modification of Graphene by Ultrasonic Tip

Into a 100 mL flask were added 0.5 g of 1,4-diaminobutane dihydrochloride and 0.5 g of 1,3-diaminopropane; the flask was adjusted to 100 mL and stirred until the amines were completely dissolved. Subsequently, 0.2 g of GNPs were dispersed and subjected to the sonication process until the mixture was homogenized.

The ultrasonic treatment was performed using a catenoidal ultrasonic probe 25 mm in diameter coupled to a home-made ultrasonic generator with an output power of 750 W, wave amplitude of 50% and variable frequency in the range of 15–50 kHz. For safety reasons, all experiments were done in a sound-abating enclosure. Three different ultrasound times (30, 45 and 60 min) were used, all treatments were done at room temperature, and at the end of the reaction time, the GNPs were washed several times with distilled water to remove unreacted amines, filtered and dried at 80 °C for 24 h. Table 1 shows the identification of the samples.

### 2.3. Characterization

All the GNPs before and after modification were characterized and analyzed by spectroscopic and microscopic techniques. Fourier-transform infrared spectroscopy (FTIR) analysis was performed in a Nicolet Nexus 670 spectrophotometer (Thermo Scientific, MA, USA), with 100 scanner and resolution of 16 cm^−1^, in the range of 400 to 4000 cm^−1^. Previously, the samples were dried in a vacuum oven at 100 °C for 15 h, and thereafter they were supported in KBr film. Scanning electron microscopy and scattered energy spectroscopy (SEM-EDS) analyses were performed in an EDAX-ESEM, XL-30-Philips instrument (Thermo Scientific, MA, USA), with an accelerating voltage of 5–25 keV. X-ray photoelectron spectroscopy (XPS) study was carried out in a K-ALPHA spectrophotometer (Thermo Scientific, MA, USA) with a monochromatic X-ray source, binding energy of 0–1350 eV, and a depth of 400 μm. Thermogravimetric analysis (TGA) was performed using a Discovery, TGA Instrument (TA Instruments, NEW, USA) from room temperature to 1000 °C with a heating rate of 5 °C/min in flowing gas consisting of 1% O_2_ and 99% Ar at a flow of 100 mL/min. The dispersion of the samples was analyzed based on the images. Modified and unmodified GNPs and solvent (8 mg and 7 mL respectively) were placed in 10-mL glass vials, and after 48 h, images were taken to show the stability of the dispersion. Hexane, ethanol and distilled water were used as solvent.

### 2.4. Adsorption of Uremic Toxins

For the determination of urea and uric acid in GNPs that were unmodified and modified with amines, the following procedure was used. Aqueous solutions of urea and uric acid at different concentrations (20, 40, 60, 80, 100, 120, 140 and 160 mg/L) were used to create calibration curves for each compound, using a UV-Vis spectrometer Shimadzu model UV-1800 (Shimadzu, Duisburg, Germany). The adsorption experiments were performed in beaker glasses of 50 mL with 20 mL of solution of 160 mg/L of concentration (urea or uric acid) and 50 mg of graphene nanoplatelets. Beaker glasses were placed in a stirring plate at 100 rpm at 37 °C for 4 h (similar duration of hemodialysis treatment). Every 30 min, an aliquot was taken, filtered and read in the UV-Vis spectrophotometer. The measurements in the UV-Vis spectrophotometer were performed in duplicate, and the experiments were carried out in triplicate. The concentration of uremic toxins in the solution was determined using the Beer–Lambert law; the maxima wavelength (λ_máx_) for urea and uric acid are 200 and 293 nm, respectively.

The removal percentage was calculated according to the Equation (1):

(1) $$\%\;\mathrm{Removal}=\frac{\left(C_{i}-C_{e}\right)}{C_{i}}\times 100$$

where *C_i_* and *C_e_* are initial and final concentrations, respectively.

The adsorption capacity of the GNPs was calculated with the Equation (2) in equilibrium:

(2) $$q_{e}=\frac{\left(C_{i}-C_{e}\right)V}{m}$$

where *V* is the volume in L of solution and m is the amount of mass in mg of absorbent.

#### 2.4.1. Adsorption Isotherm

The Langmuir and Freundlich models were used to describe the adsorption equilibrium data. For assessment of both models, the absorption isotherms data were fitted and the correlation coefficient (*R*^2^) was calculated using the trendline command in Microsoft Excel.

The Langmuir isotherm was calculated by the Equation (3):

(3) $$\frac{C_{e}}{q_{e}}=\frac{C_{e}}{q_{m}}+\frac{1}{K_{L}q_{m}}$$

where *q_e_* (mg⋅g^−1^) and *C_e_* (mg⋅L^−1^) are the concentrations of the solid and liquid phases of adsorbate in equilibrium, respectively, *q_m_* is the maximum adsorption capacity, and *K_L_* is the constant obtained from the graph of *C_e_*/*q_e_* against *C_e_*.

The Freundlich isotherm was calculated by the Equation (4):

(4) $$\ln q_{e}=\ln K_{F}+\left(\frac{1}{n}\right)lnC_{e}$$

where *K_F_* (mg⋅g^−1^) (L⋅mg^−1^) and 1/*n* are the Freundlich constants related to the adsorption capacity and *n* is the heterogeneity factor calculated by linearly plotting *ln*⋅*q_e_* against *ln*⋅*C_e_*.

#### 2.4.2. In Vitro Blood Compatibility

The results of hemolysis assays with the modified graphene with amine were not cytotoxic when using 500 mg/mL, and less than 5% hemolysis according to the E2524-08. Therefore, these materials can be used for in vivo applications such as hemodialysis. Wang et al. describe the hemolysis values above the negative control as falling into three classifications: (1) 0–2%, which represents non-hemolytic, (2) 2–5%, slightly hemolytic, and (3) > 5%, hemolytic [28].

## 3. Results and Discussion

### 3.1. Fourier Transform Infrared Spectroscopy (FTIR)

**Figure 1:**
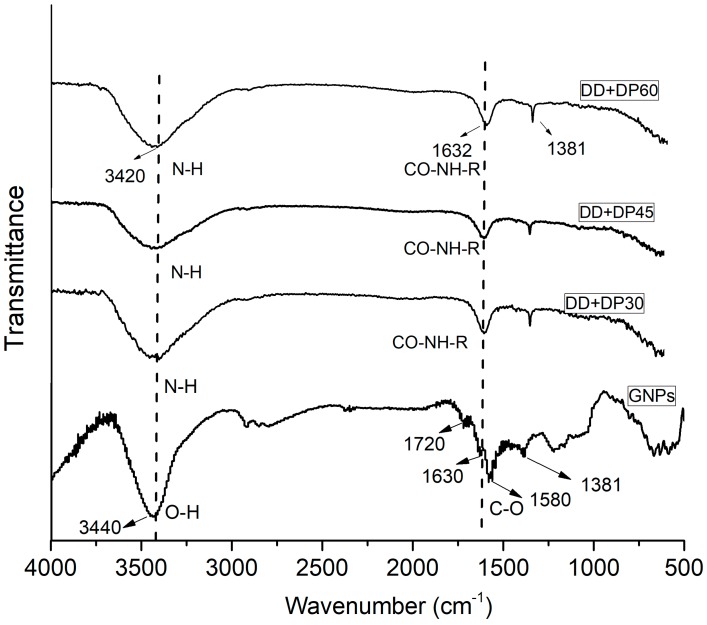
FTIR spectra of GNPs, DD+DP30, DD+DP45 and DD+DP60.

Figure 1 shows the infrared spectra of the GNPs and the modified GNPs with the amine mixture treated with ultrasonic waves of different amplitudes and frequencies for different times.

The FTIR spectrum of GNPs without any treatment shows broad bands at 3550, 1740, 1630 and 1050–1220 cm^−1^, corresponding to hydroxyl (OH), carboxyl (COOH), carbonyl (C=O) and epoxy groups. These bands confirm the presence of chemical groups that contain oxygen on the surface of the graphene [29,30]. The modified GNP spectra show fewer bands with respect to the unmodified GNPs; they do not show bands corresponding to the carboxylic acids and epoxides, which suggests a reaction with the amino group and formation of C-N bonds on the surface of the GNPs. The FTIR spectra show a wide and intense band at 3420 cm^−1^ characteristic of N-H stretching, the presence of -OH and N-H groups in graphene oxide has been reported previously [31,32]. Another band located at 1632 cm^−1^ with less intensity indicates the presence of amide groups [12,32], the small band at 1381 cm^−1^ remains unchanged, and corresponds to the stretching vibration peak –C-H (in-plane). The FTIR spectra confirm the covalent bond formation between the neat GNPs and the functional groups of the diamines.

One might think that a longer exposure period of ultrasonic would lead to greater modification of the surface, but it should also be considered that, at long periods of exposition, fragmentation of the nanoparticles, shear forces and cavitation occur [33].

The modified GNP spectra are similar, but DD+DP30 showed the most intense bands, which can be attributed to major functionalization in the GNP surface with a reaction time of 30 min; this spectroscopic evidence was confirmed by TGA analysis.

### 3.2. X-Ray Photoelectron Spectroscopy (XPS)

**Figure 2:**
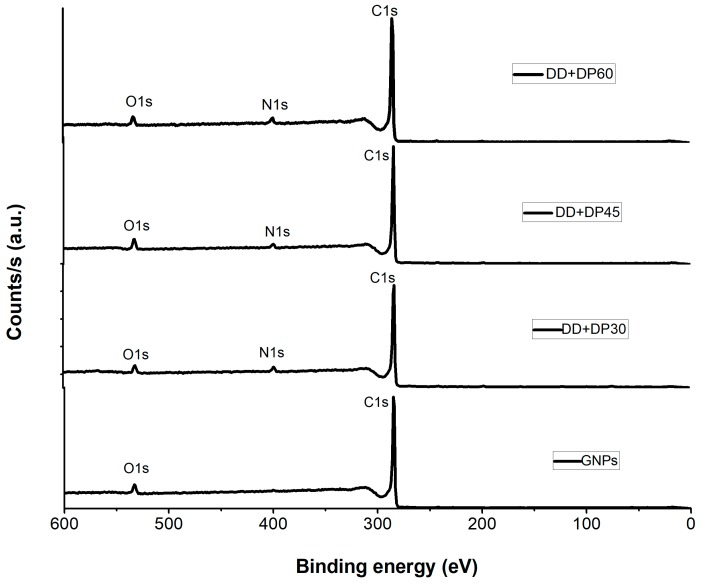
XPS spectra of graphene, DD+DP30, DD+DP45 and DD+DP60 samples.

XPS serves to penetrate the first 10 nm of a surface, identifying all the elements present (except H, He) in concentrations greater than 0.1%. The XPS spectra of the samples of GNPs DD+DP30, DD+DP45 and DD+DP60 are shown in Figure 2. In the spectrum of the unmodified samples (GNPs), only the signals corresponding to C1s and O1s can be observed, at 284.92 and 532.89 eV, respectively; similar displacements have been reported for graphene oxide reduction using ultrasound-assisted reaction, which were localized at 284.5 eV for the C1s and around 530 eV for the O1s [34].

Based on quantitative analysis, Table 2 shows the values of the atomic percentages obtained, the GNP sample showed 95.85% for C1s and 4.15% for O1s. For the DD+DP30, DD+DP45 and DD+DP60 samples, in addition to the C1s and O1s signals, the signal for N1s at bond energies of 399 eV was present in different atomic percentages, corroborating the functionalization of the GNPs. The most intense signals were observed for sample DD+DP30. The application of high-frequency sound waves could provide an effective energy source for modifying the surface of GNPs in a short time and improving dispersion in different media [35].

Jun et al., in 2015, when producing a graphene hybrid material for obtaining methane, reported nitrogen signals at the same binding energy values of 398.6 and 399.8 eV, corresponding to sp^2^ hybridized aromatic N bonded to carbon atoms (C=N-C) and the tertiary N groups [N-(C_3_)], respectively [36]. Table 2 shows the characteristic signals of photoemitted electrons and the atomic percentages corresponding to each of the samples. The results indicate that DD+DP30 contains the highest amount of nitrogen. Samples treated with ultrasonic radiation for longer than 30 min exhibited a lower degree of functionalization because the ultrasound is able to generate drastic reaction conditions that can destroy the chemical groups on the surface of the GNPs and generate structural damage [37]. 

**Figure 3:**
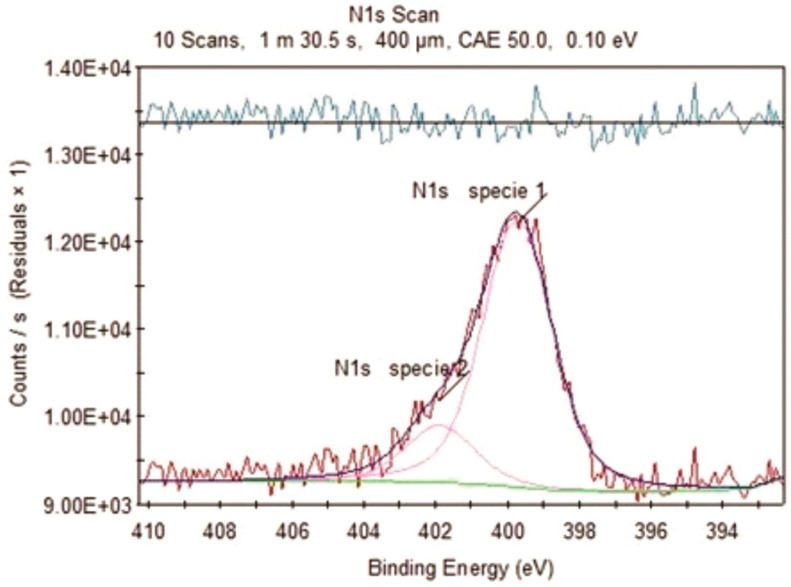
N1s deconvolution peak of the DD+DP30 sample.

To corroborate the chemical environment, deconvolution of the N1s signal of the DD+DP30 sample was performed, because a stronger signal was seen through XPS in this sample. The N1s deconvolution spectrum (Figure 3) presents two peaks at 399.72 and 401.88 eV, the first was assigned to non-protonated nitrogen (typical of amide groups), while the second corresponds to protonated nitrogen (^+^NH_3_). A similar spectrum was reported in the formation of amides by the reaction of carboxylate function and cationic amine function [38]. The XPS results are in agreement with the evidence of FTIR, where the sample modified for 30 min has the highest degree of functionalization and the functional groups present are amides and cationic amines, the presence of aromatic and tertiary nitrogen atoms was discounted.

### 3.3. Scanning Electron Microscopy (SEM-EDS)

**Figure 4:**
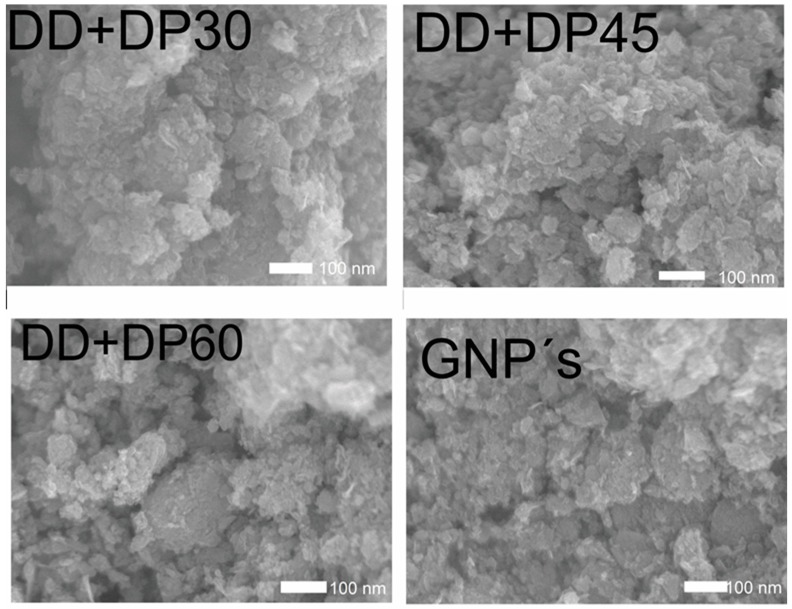
SEM images of graphene, DD+DP30, DD+DP45 and DD+DP60.

To observe the surface morphological change and the chemical elements present, Scanning Electron Microscopy and Energy Dispersive Spectroscopy studies were performed. Figure 4 shows the SEM images for the GNPs, DD+DP30, DD+DP45 and DD+DP60 samples. All micrographs exhibit similar morphologies; the surface modifications cannot be seen with the resolution used. Despite this, we are able to observe the agglomerates formed in each sample. The most important difference was found in the DD+DP30 sample, which shows larger-sized agglomerates than the other samples. Nevertheless, the SEM-EDS technique provides relevant information regarding the chemical composition of the samples.

**Figure 5:**
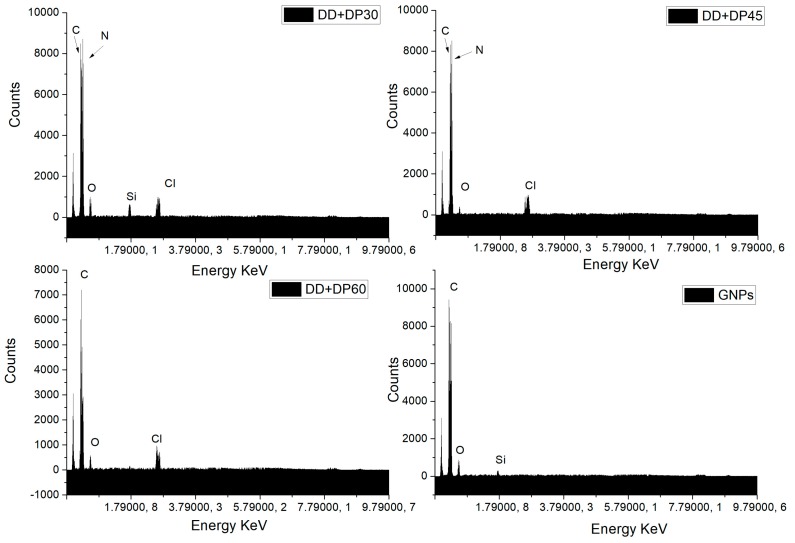
EDS spectra of GNPs, DD+DP30, DD+DP45 and DD+DP60.

**Figure 6:**
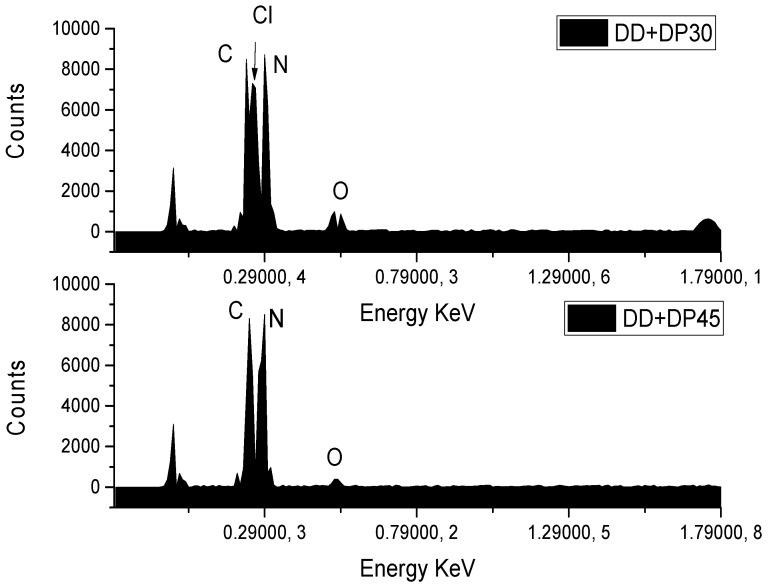
Amplification of EDS spectra of DD+DP30 and DD+DP45.

The EDS spectra for the samples DD+DP30, DD+DP45, DD+DP60 and GNPs (Figure 5) show signals corresponding to the following elements: carbon (C), oxygen (O), nitrogen (N) and chlorine (Cl). The assignment of these peaks is in agreement with other studies [39,40,41], which reported that the spectrum of GNPs only exhibits signals of C and O [39]. While modified GNPs spectra exhibit signals of all the elements indicated, the DD+DP60 spectrum does not show the nitrogen assignment; however, the Cl signal implies the presence of nitrogen. Amplification of the spectra in the region 0–179,000 KeV was performed for the samples with the highest N content in order to correctly identify the signals of C, N and Cl (Figure 6); the chlorine signal present in DD+DP60 suggests a higher content of protonated nitrogen (RNH_3_^+^ Cl^−^).

### 3.4. Thermogravimetric Aanalysis (TGA)

**Figure 7:**
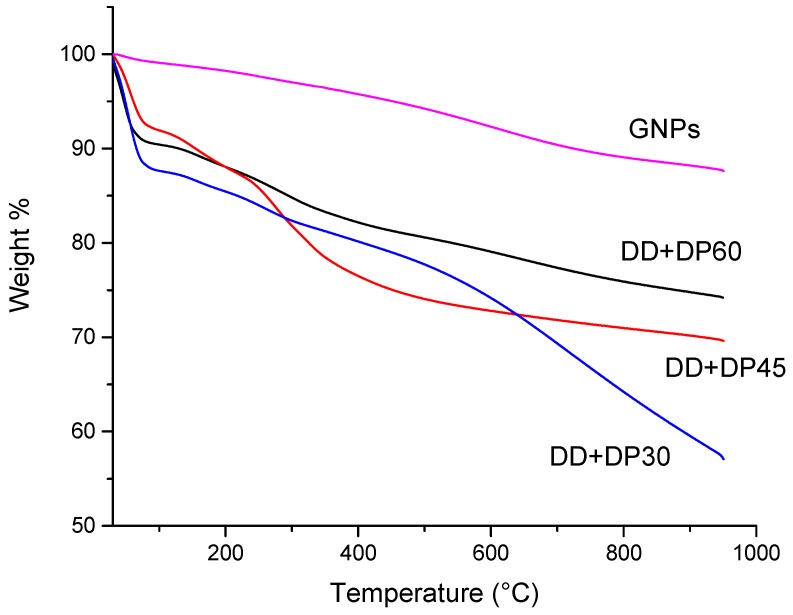
TGA of GNPs and modified graphene.

The thermogravimetric analysis of GNPs before and after surface modification are shown in Figure 7. The unmodified GNPs exhibit a total weight loss of 12.5% at a temperature of 950 °C, the weight loss at a temperature of 100 °C was 0.9%, which may be associated with water molecules on the surface of the GNPs.

The modified graphene thermograms presented a different thermal behavior; the functionalization of the surface of GNPs causes a lower thermal stability and makes the surface more hydrophilic. Weight loss at a temperature of 100 °C has values of 8.1–12.6%, which indicates that the modified graphene has a higher water adsorption capacity than unmodified GNPs, because the functional groups favor the interaction with water molecules through the formation of hydrogen bonds (-N ---- H).

On the other hand, Table 3 shows the loss of weight at different temperatures; it can be seen that the sample DD+DP30 has the highest values at all temperatures, which confirms the greater degree of functionality of the surface of the GNP.

### 3.5. Dispersion Test

The dispersion test in different solvents provided information on the modification of the samples. Dispersion tests were performed using the unmodified GNPs as a control. In Table 4, we can observe vials of all samples (GNPs, DD+DP30, DD+DP45 and DD+DP60) dispersed by ultrasound for 48 h, different solvents were used, such as distilled water, ethanol and hexane.The GNPs without treatment by ultrasound were deposited in the bottom of the vial, confirming their low capacity to interact with the different solvents. However, for the three samples treated with ultrasound with ethanol and hexane, the particles remained quite dispersed after 48 h of being dispersed; this behavior shows that there is a high interaction between the molecules of the solvents and the functional groups present in the treated GNPs [42].

In distilled water, the sample that presented the best dispersion was that treated for 45 min, since it remained stable after 48 h. The DD+DP30 and DD+DP60 samples were adequately dispersed at the beginning of the test and after 48 h, a portion of the particles were deposited on the bottom, while others were suspended; this can be attributed to the time of modification not being optimal for the treatment of GNPs.

The dispersion in distilled water of the modified GNPs was only favorable for the DD+DP45 sample, the same situation was observed in ethanol and hexane, the nanoparticles remained in suspension after 48 h of being dispersed, this can be attributed to the presence of alkyl chains of the amines used for the modification.

### 3.6. Proposal for Surface Modification in Graphene Platelets

**Figure 8:**
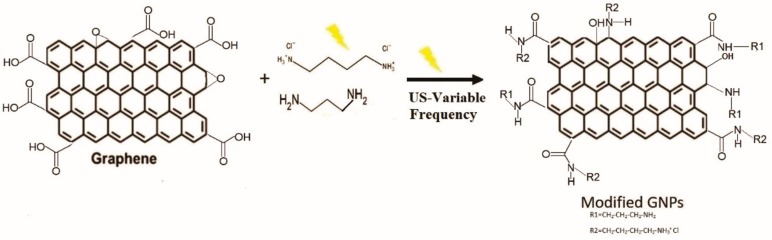
Proposed scheme for the chemical modification of GNPs with ultrasound waves.

The analysis of the results obtained by FTIR, XPS, SEM and TGA leads us to confirm the presence of different functional groups on the surface of GNPs (Figure 8). The amines used contain two functional groups that are able to react with a platelet or several platelets; for simplicity, only the reaction of a functional group is illustrated.

The cavitation generated by the ultrasonic horn under the influence of ultrasound waves of different amplitudes and frequencies, as well as the fluctuation of the pressure, help to carry out the chemical modification and to activate the surfaces of the GNPs. Defects in the edges of the graphene represent the most reactive areas of the material.

### 3.7. Uremic Toxin Adsorption

**Figure 9:**
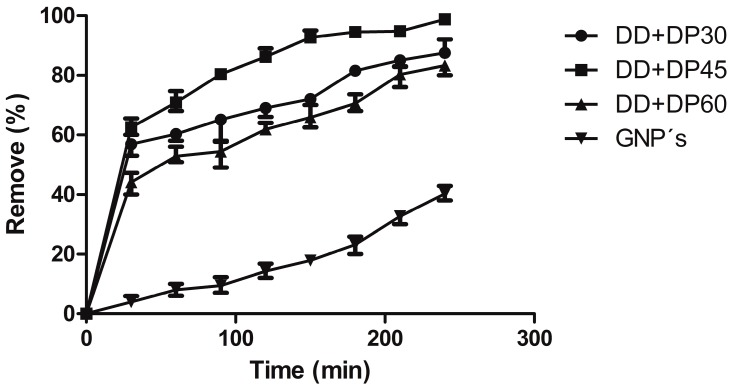
Urea removal percentage for GNPs and modified GNPs.

The adsorption models consider data points within a period of 4 h (the time required for the hemodialysis treatment). We aimed to evaluate the absorption of toxins and the behavior of the different modifications made over GNPs and the unmodified GNPs. The results of the removal percentage of urea from the 160 mg/L solution prepared with distilled water are presented in Figure 9. Unmodified graphene has a 42% removal of urea, while modified graphene samples reach values of 79–97%; the maximum percentage of removal was obtained for the DD+DP45 sample, which presented a 97% removal of urea. 

Modified graphene with amines showed an excellent adsorption capacity of urea; this was more effective for removing the urea in comparison to modified graphene with organic acid, as reported recently [14]. This can be attributed to the better interaction between the urea and the nitrogen atoms present on the GNP surface and the high percentage achieved by the variable frequency ultrasonic generator. The modifie GNPs in this investigation can be considered effective absorbent materials, and as an environmentally friendly and economically viable adsorbent for removing toxins.

**Figure 10:**
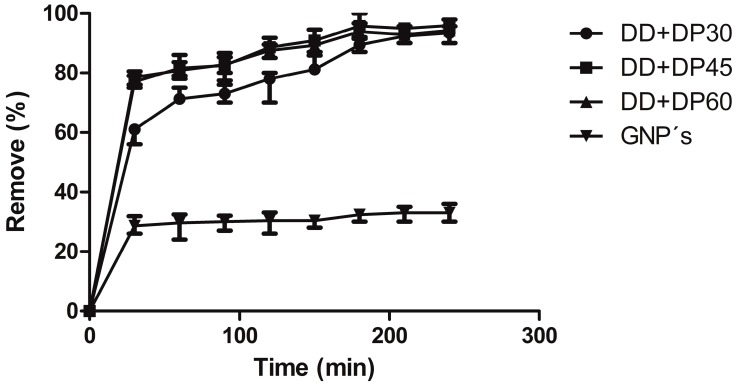
Removal percentage of uric acid for GNPs and modified GNPs.

The removal percentage of uric acid is presented in Figure 10, which for GNPs show a value of about 42.9%; conventional dialysis systems (polyethersulfone or cellulose membranes) have similar removal percentages of uremic toxins. For the three samples containing modified GNPs with amines, up to 95% of uric acid could be eliminated, in turn making this a promising material for use in hemodialysis. Modified graphene with amines was found to be more effective than modified graphene with carboxylic acids, which only has values of 30–50% removal. Again, we attribute this to the high percentage of functionalization with nitrogenous groups [14].

**Figure 11:**
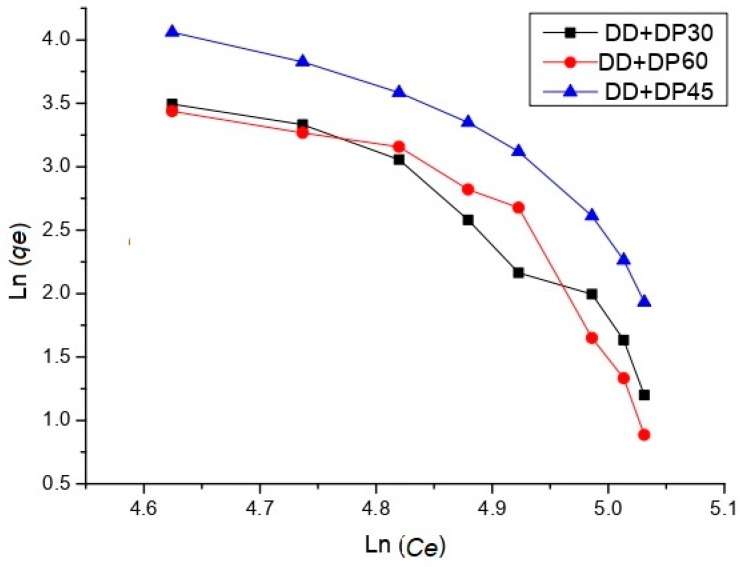
Freundlich model of adsorption for urea of GNP and modified GNP samples.

Table 5 shows the results of urea adsorption on GNPs samples. The values of the correlation coefficient of unmodified GNPs indicate a monolayer adsorption (Figure 11), as has already been reported by Wang *et al.* for the adsorption of heavy metals in graphene [43]. In contrast, the samples of amine-modified graphene show values that indicate that multilayer adsorption is being carried out. The Freundlich isotherm assumes a heterogeneous adsorption process that occurs on the surface of the adsorbent; in this case, the GNPs are a low-cost alternative to nanoparticles in relation to graphene nanosheets or monolayer graphene, since the latter have the high price of amine-modified graphene, which allows the adsorption of the uric acid in several layers. This model for the adsorption of creatinine in activated carbon was adjusted by Cao et al. [44].

The Langmuir isotherm is adjusted for the unmodified graphene; therefore, there is monolayer adsorption. Similar results for the adsorption of creatinine and urea have been reported for graphene oxide [45]. The samples of amine-modified graphene fit the Freundlich model; the adsorption process is assumed to be heterogeneous (Table 6).

**Figure 12:**
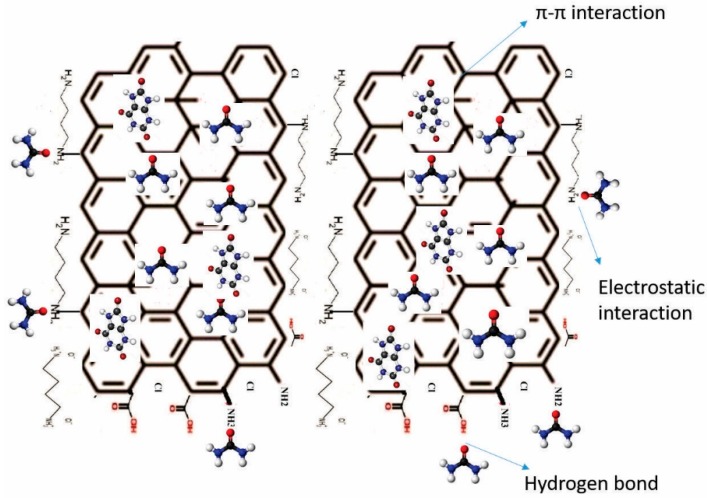
Proposed scheme for urea and uric acid adsorption onto the modified GNPs.

The Freundlich isotherm proposes a multilayer adsorption. Figure 12 shows two sheets of modified graphene with amino groups, in which the urea and uric acid molecules are adsorbed by electrostatic interactions, hydrogen bonding and π-π interactions.

### 3.8. Hemolysis Assay

**Figure 13:**
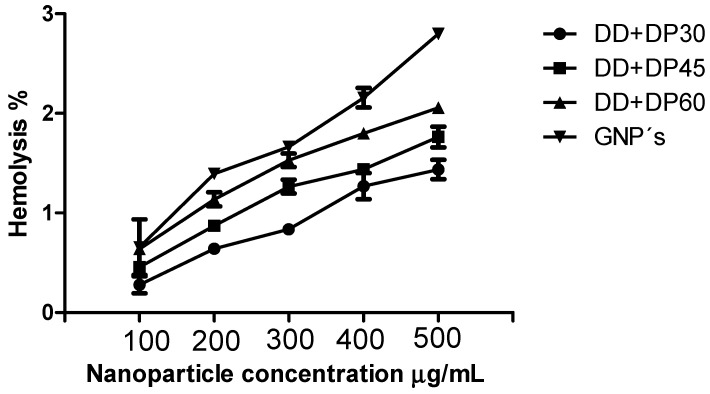
Hemolysis curves of samples unmodified graphene and graphene modified with amines at concentrations of 100, 200, 300, 400 and 500 mg/mL using DPBS as the negative control (0% hemolysis) and PEG as the positive control (100% hemolysis).

The application of nanoparticles in hemodialysis treatment involves contact with blood cells, although they are in a polymeric membrane. Figure 13 shows a hemolysis percent af 100–500 mg/mL for each sample. For unmodified graphene, this is 2.8% at 500 mg/mL, a result which suggests that the material is non-hemolytic. The E2524-08 protocol indicates that at more than 5% hemolysis, the material will cause damage to red blood cells [46]. The samples DD+DP30, DD+DP45 and DD+DP60 exhibited a lower percentage of hemolysis (1.3–1.7%) compared with unmodified graphene; similar percentages were reported by Shanti et al. They obtained 1.7% hemolysis for the functionalized carbon dots [11]. 

Hemolysis curves are illustrated in Figure 13, where the hemolytic effect can be observed at different concentrations, a low percentage of hemolysis was obtained when the graphene was modified; similar results were reported by Wu et al. The evaluation of modified graphene oxide with hyaluronic acid led to a material with good blood compatibility [12]. Therefore, the amine-modified graphene is biocompatible with blood cells, and this material can be incorporated into a polymeric membrane for use in the treatment of hemodialysis. 

The new materials developed in the present investigation are good candidates for use in CKD; therefore, detailed study on their reusability and compatibility with polymeric membranes should be carried out.

## 4. Conclusions

According to the results, the surface modification of GNPs with amine mixtures (1,4-diaminobutane dihydrochloride and 1,3-diaminopropane) under the influence of ultrasound waves of different amplitudes and frequencies was carried successfully out. 

The reaction of hydrophilic amine groups with carboxylic acids and epoxides present on the surface of GNPs proceeds by amidation and ring opening reactions. The functionalization was confirmed by FTIR, XPS, SEM-EDS and TGA. The functionalization of GNPs leads to a surface with greater hydrophilic character and improves its dispersion capacity in water, ethanol and hexanes.

With respect to the study of the adsorption of uremic toxins, the percentage of removal was excellent, with sample DD+DP45 achieving a 97% adsorption of urea and uric acid. The values obtained were superior to conventional materials, which only achieve 60% elimination of uremic toxins.

These results are attributed to the chemical modification of the surface of the GNPs, in addition to the interaction between the chemically modified nanoparticles and the physisorption process of the toxins evaluated. This investigation contributes to the absorption of uremic toxins using modified GNPs, the method of modification with ultrasound at variable frequency, can be considered as environmentally friendly and economically viable, so that these new materials are an excellent alternative for integration into filters of non-woven fabric for use in CKD.

## Figures and Tables

**Table 1:** Identification and reaction time of the GNPs modified.

Sample	Reaction Time (min)
GNPs	0
DD+DP30	30
DD+DP45	45
DD+DP60	60

**Table 2:** XPS data in terms of binding energies and atomic percent.

Sample	C1s Peak (eV)	O1s Peak (eV)	N1s Peak (eV)	C1s At %	O1s At %	N1s At %
GNPs	284.92	532.89	ND	95.85	4.15	ND
DD+DP 30	284.45	532.42	399.43	91.62	4.15	4.23
DD+DP 45	284.29	532.36	399.38	90.46	6.23	3.31
DD+DP 60	284.97	532.81	399.9	92.34	4.26	3.4

(ND) Not detected; (At%) Atomic percent; (eV) Electronvolts

**Table 3:** TGA data of GNPs and modified GNPs.

Sample	Weight Loss at 100 °C (%)	Weight Loss at 250 °C (%)	Weight Loss at 500 °C (%)	Weight Loss at 750 °C (%)	Residue at 950 °C (%)
GNPs	0.9	2.35	5.7	10.2	12.5
DD+DP30	12.6	16.0	25.9	33.2	42.9
DD+DP45	8.1	14.1	22.2	28.6	30.4
DD+DP60	9.6	13.4	19.6	23.6	25.8

**Table 4:** Dispersion tests (for 48 h) in different solvents for unmodified and modified GNPs.

Solvent	GNPs	DD+DP30	DD+DP45	DD+DP60
Distilled water	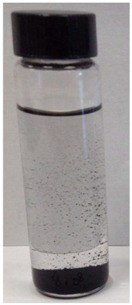	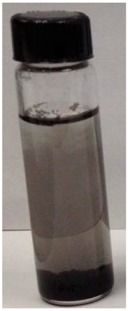	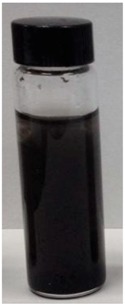	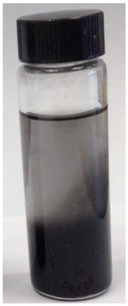
Ethanol	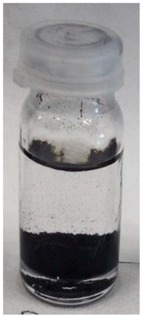	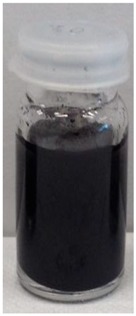	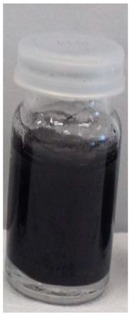	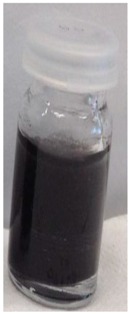
Hexane	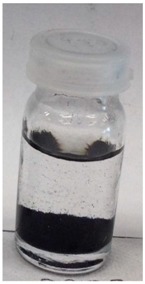	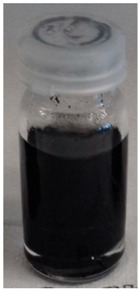	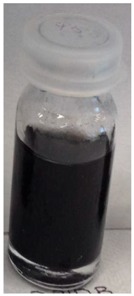	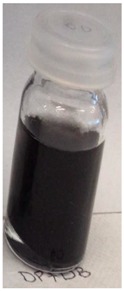

**Table 5:** Parameters of the isotherm constants and correlation coefficients calculated for urea adsorption.

Sample	Langmuir			Freundlich		
	k	q_max_	R^2^	n	K_f_	R^2^
GNPs	0.010	0.056	0.973	1.050	1.189	0.6769
DD+DP30	0.118	10.20	0.8875	2.90	17.47	0.9461
DD+DP45	0.556	63.13	0.6977	6.23	33.49	0.8768
DD+DP60	0.075	5.46	0.9002	2.11	13.71	0.9401

**Table 6:** Parameters of the isotherm constants and correlation coefficients calculated for the adsorption of uric acid.

Sample	Langmuir			Freundlich		
	k	q_max_	R^2^	n	K_f_	R^2^
GNPs	1.17	0.001	0.9996	0.009	0.007	0.9993
DD+DP30	0.343	37.08	0.7335	5.02	27.58	0.8902
DD+DP45	1.11	144.17	0.6877	10.04	52.37	0.91
DD+DP60	1.58	210.05	0.6459	11.57	59.98	0.8911
